# Utilizing Network Toxicology and Molecular Dynamics Simulations to Efficiently Evaluate the Neurotoxicity and Underlying Mechanisms of the Endocrine-Disrupting Chemical Triclosan

**DOI:** 10.3390/ijms26199458

**Published:** 2025-09-27

**Authors:** Hao Wang, Yunyun Du, Jin Ji, Chunyan Wang, Zexin Yu, Xianjia Li, Yueyi Lv, Suzhen Guan

**Affiliations:** Ningxia Key Laboratory of Environmental Factors and Chronic Disease Control, School of Public Health, Ningxia Medical University, Yinchuan 750004, China; 18615217356@163.com (H.W.); dyy202323@163.com (Y.D.); j637256869@163.com (J.J.); wangcy_0225@163.com (C.W.); zexin_yu111@163.com (Z.Y.); 13466884661@163.com (X.L.); 15091470948@163.com (Y.L.)

**Keywords:** triclosan, network toxicology, neurodevelopmental disorders, molecular docking, molecular dynamics simulation

## Abstract

This study aims to elucidate the neurodevelopmental toxicity and molecular mechanisms of endocrine-disrupting chemicals (EDCs) in neurodevelopmental disorders (NDDs) through a network toxicology approach, using triclosan exposure as a case example. Potential targets of triclosan were identified via comparative analysis of toxicogenomics databases such as the Comparative Toxicogenomics Database (CTD), Similarity Ensemble Approach (SEA), SwissTargetPrediction, and TargetNet. NDD-related targets were retrieved from GeneCards, Disease Gene Network (DisGeNET), and Online Mendelian Inheritance in Man (OMIM), resulting in 633 overlapping genes associated with disease pathology and triclosan effectors. Protein–protein interaction networks were constructed using STRING and Cytoscape, applying median-based algorithms to identify six core genes: *AKT1*, *TP53*, *EGFR*, *FN1*, *SRC*, and *ESR1*. Gene Ontology (GO) and Kyoto Encyclopedia of Genes and Genomes (KEGG) enrichment analyses via Metascape revealed that triclosan-induced NDDs are primarily associated with endocrine signaling disruption and activation of the PI3K-Akt pathway. Molecular docking with CB-Dock2 demonstrated strong binding affinities between triclosan and the core targets, while YASARA molecular dynamics simulations confirmed stable interactions, notably with EGFR, exhibiting high binding stability. Collectively, these findings delineate the potential molecular mechanisms underlying triclosan-induced NDDs and underscore the utility of network toxicology, molecular docking, and molecular dynamics simulations in assessing neurotoxicity and related molecular pathways. This research provides novel insights for future investigations, enhances understanding of the potential impact of neurodevelopmental disorders on health, and lays a scientific foundation for the development of preventive and therapeutic strategies.

## 1. Introduction

Triclosan (TCS), as a broad-spectrum antimicrobial agent, has been extensively incorporated into commercial and healthcare formulations such as soaps, dentifrices, hand sanitizers, cosmetics, textiles, and plastics owing to its potent bactericidal and bacteriostatic properties [1]. In 2012, the World Health Organization (WHO) designated triclosan as an emerging environmental endocrine disruptor, raising concerns regarding its potential endocrine-disrupting effects and associated health risks [2]. Subsequently, in 2016, the U.S. Food and Drug Administration (FDA) prohibited the inclusion of triclosan in over-the-counter consumer antiseptic products while permitting its use within medical and clinical settings [3]. Nonetheless, the widespread deployment of disinfectants and hand hygiene products during the COVID-19 pandemic has intensified triclosan accumulation in environmental matrices [4], complicating its environmental fate and transport. The synergistic interactions between triclosan and co-occurring pollutants pose additional challenges to environmental safety assessments [5,6]. Epidemiological and toxicological studies indicate that chronic exposure to triclosan-containing dentifrices correlates with elevated plasma concentrations [7], and its frequent detection in maternal biological samples—including urine, blood, breast milk, amniotic fluid, and neural tissues—raises concerns about transplacental transfer and neurodevelopmental risks to the fetus via the blood–brain barrier [8,9,10].

Neurological developmental disorders (NDDs), such as autism spectrum disorder (ASD) and attention deficit hyperactivity disorder (ADHD), may result from teratogenic exposures during gestation and are modulated by a complex interplay of genetic predisposition, epigenetic alterations and environmental factors [11,12]. Current research on triclosan toxicity primarily focuses on acute toxicity and reproductive developmental toxicity, with limited investigations into its impact on human neurohealth; existing studies provide preliminary epidemiological evidence of potential adverse effects. The neurotoxicity of triclosan is closely associated with dysregulation of multiple signaling pathways due to its endocrine-disrupting properties [13,14]. Initially, at the nuclear receptor level, triclosan interferes with thyroid hormone homeostasis by competitively binding to TR, thereby affecting critical processes in neurodevelopment [15]. Furthermore, triclosan acts on GPER to activate downstream pathways PKC/MAPK, leading to the upregulation of miR-144, which causes neurodevelopmental toxicity and abnormal motor behavior in zebrafish larvae [16]. Triclosan also targets multiple non-nuclear receptor targets. Animal experiments have shown that triclosan regulates the Nrf2/HO-1 pathway through the PI3K/Akt/JNK signaling cascade, increasing intracellular ROS production and upregulating the expression of pro-apoptotic proteins, thus inducing neuronal oxidative damage [17]. Concurrently, chronic maternal exposure to triclosan can induce neurodevelopmental disorders in offspring by mediating increased peripheral inflammation and hippocampal neuronal synaptic damage, leading to autism-like social behavioral deficits [18,19]. Moreover, triclosan may activate microglia, increase PKM2 dimerization, and mediate STAT3 phosphorylation, resulting in behavioral disorders and neurotoxicity in offspring [20]. Epidemiological studies further support these mechanisms, showing a positive correlation between prenatal triclosan exposure levels in pregnant women and the risk of early childhood language development delays and attention deficit symptoms. Additionally, there is a dose-dependent association between the concentration of triclosan metabolites in children’s urine and the severity of core autism symptoms [21]. Notably, pediatric exposure levels surpass those of adults, compounded by the immature blood–brain barrier in children, rendering them more susceptible to triclosan-induced neurotoxicity [22]. Nonetheless, the precise molecular targets involved in triclosan-induced neurodevelopmental disorders and transgenerational epigenetic modifications remain to be elucidated; comprehensive mechanistic studies are essential to establish a scientific foundation for early environmental intervention strategies.

This study employed network toxicology approaches to construct complex regulatory networks, elucidating the multifaceted toxicological pathways by which triclosan exacerbates neurodevelopmental disorders. It aims to identify potential pathogenic mechanisms, offering strategic insights for environmental pollutant toxicity assessment, and providing guidance for regulatory policies and the rational application of antimicrobial agents.

## 2. Results

### 2.1. Target Identification

**Figure 1:**
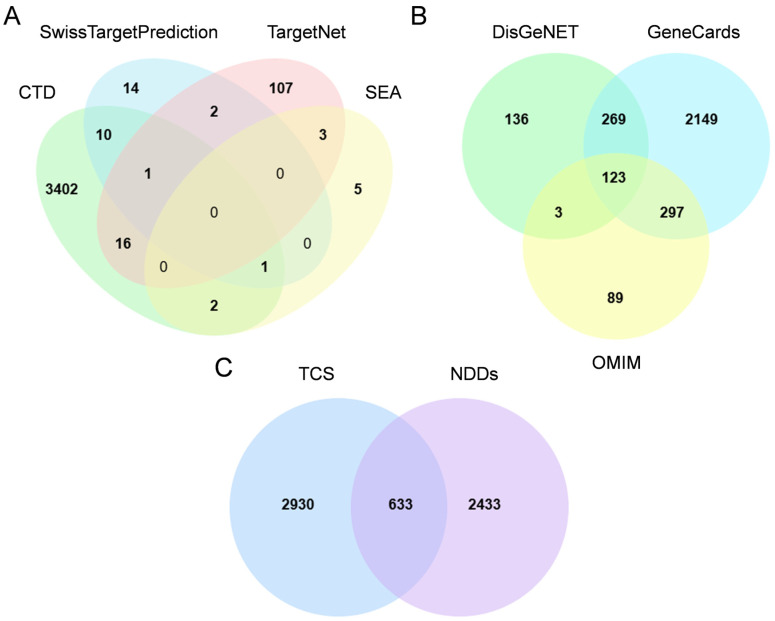
Target identification workflow. (**A**) Target collection for triclosan (TCS), including 3563 targets retrieved from Comparative Toxicogenomics Database (CTD), SwissTargetPrediction, Similarity Ensemble Approach (SEA), and TargetNet; (**B**) target collection for neurodevelopmental disorders (NDDs), including 3066 targets retrieved from GeneCards, Disease Gene Network (DisGeNET) and Online Mendelian Inheritance in Man (OMIM); (**C**) Venn diagram showing intersecting targets between TCS and NDDs.

A total of 3563 triclosan target genes were identified through integration of data from the CTD, SwissTargetPrediction, SEA, and TargetNet databases (Figure 1A). Additionally, NDD targets were extracted from GeneCards, DisGeNET and OMIM, yielding 2838, 531, and 512 targets, respectively. Deduplication, defined here as the removal of duplicate gene symbols across the three databases to retain unique entries, resulted in 3066 unique targets (Figure 1B). The intersection between disease-related genes and triclosan targets revealed 633 shared genes (Figure 1C), indicating a significant association with triclosan-induced neurodegenerative disorders. Detailed information on each gene set in the Venn Diagram are presented in Supplementary Table S1.

### 2.2. Core Genes of Triclosan-Induced Neurodevelopmental Disorder Shared Gene Network

**Figure 2:**
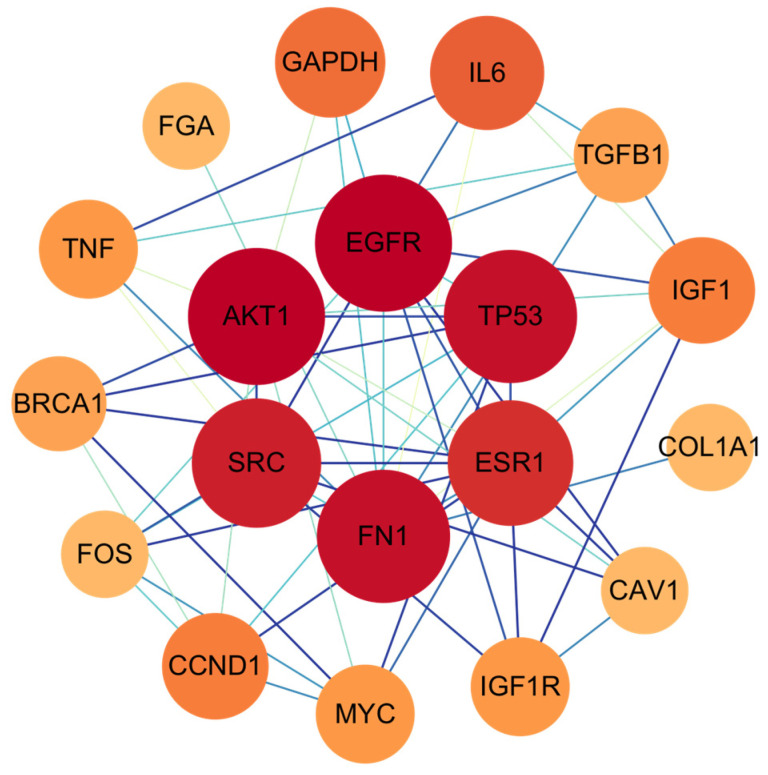
The protein-protein network interactions in core targets. The network diagram represents complex interactions among 19 core genes, highlighting their functional connections. Node color (red to orange gradient) and node size (large to small gradient) correspond to the degree value: red/larger nodes represent genes with higher degree values, while orange/smaller nodes represent genes with lower degree values.

Following the identification of 633 intersecting genes between triclosan and NDDs, we investigated the potential functional interactions of these genes at the protein level. To this end, a PPI network was constructed using STRING, encompassing all 633 gene products. The resulting network comprised 632 nodes, 985 edges, and an average node degree of 3.12. Following the import of a TSV file generated from STRING into Cytoscape 3.10.0, network topology analysis was performed using the CytoNCA plugin. Employing the median of six topological parameters as a threshold, genes exhibiting parameter values exceeding the median were retained. This approach allows for a comprehensive assessment of node importance from multiple perspectives, ensuring the robust identification of core genes. This process culminated in the identification and visualization of the top 19 hub genes: *CAV1*, *COL1A1*, *SRC*, *TGFB1*, *IGF1*, *GAPDH*, *TNF*, *BRCA1*, *ESR1*, *MYC*, *AKT1*, *IL6*, *FN1*, *EGFR*, *IGF1R*, *FOS*, *FGA*, *CCND1*, and *TP53* (Figure 2).

### 2.3. Validation of the Core Gene Random Dataset

**Figure 3:**
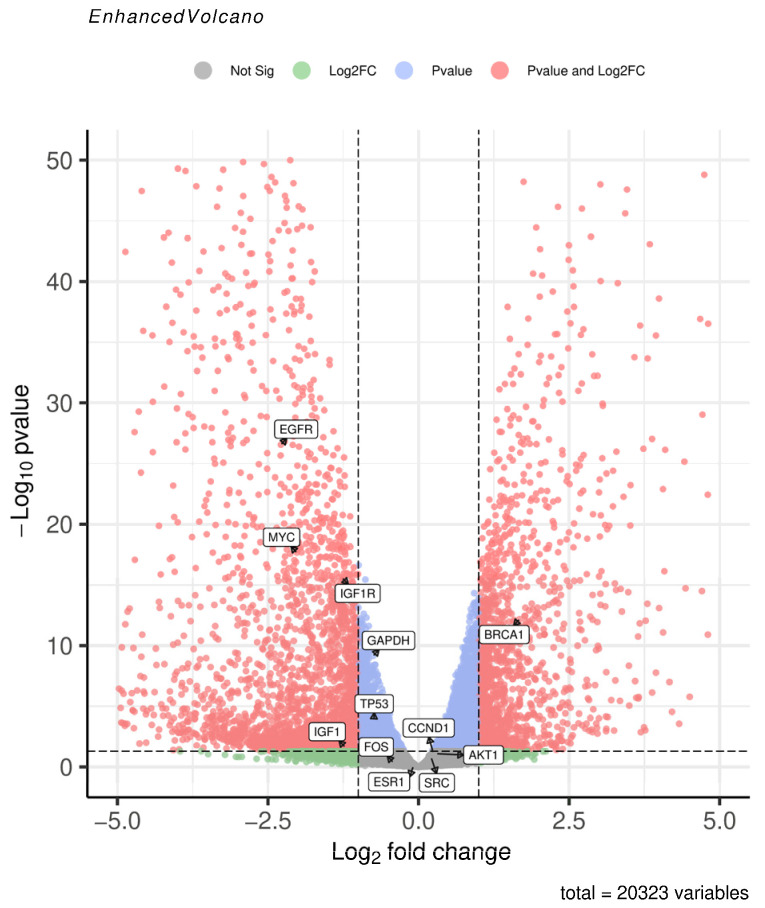
Volcano plot of the core genes detected and differentially expressed in GSE230714. Significant hits are shown in red: |log_2_FC| ≥ 1 and *p* value < 0.05. Positive log_2_FC (right side): up-regulated in MECP2-KO (case) neurons; Negative log_2_FC (left side): down-regulated in MECP2-KO neurons.

To validate the core gene expression profiles, we randomly selected the GSE230714 dataset. This dataset includes samples of human embryonic stem cell (hESC)-derived MECP2 wild-type (WT, control group) and MECP2 mutant (KO, simulating NDD subtype RTT pathological state, case group) neurons, with three biologically independent samples in each group. ERCC RNA Spike-in, an external RNA mixture of known concentration, was added to the experiment to correct for differences in cell number input and ensure the standardized accuracy of gene expression data. We performed differential expression analysis of the control and case groups of the dataset using the GEO2R online analysis tool. Visualization of differential expression results was performed via volcano plots on the microarray analysis platform. The results indicate that among the 19 selected core genes, only 12 were present in the gene expression matrix of the dataset, while the remaining 7 were absent. Consequently, the volcano plot only displays these 12 genes that are present in the dataset. Notably, BRCA1 was significantly upregulated in the case group. EGFR, MYC, IGF1R, and IGF1 were significantly downregulated. GAPDH, TP53, and CCND1 exhibited minor expression changes, yet these changes were statistically significant (Figure 3).

### 2.4. Enrichment Analyses for GO Terms and KEGG Pathways

**Figure 4:**
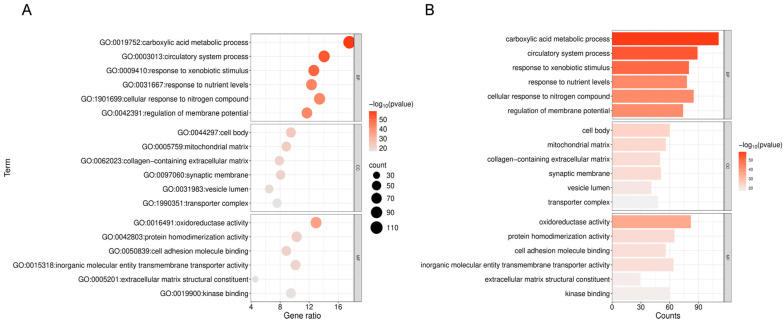
Enrichment analysis of GO for 633 shared genes. (**A**) The size of each bubble corresponds to the gene expression level within a specific pathway. “GO:XXXXXXX” is the official Gene Ontology identifier. Gene ratio: the proportion of genes in the set that share the term. (**B**) The bar chart illustrates the top six enriched GO categories—biological process (BP), cellular component (CC), and molecular function (MF)—with the lowest FDR values among 633 shared genes.

To elucidate the biological mechanisms by which triclosan exacerbates NDDs, GO and KEGG enrichment analyses were performed on 633 shared targets using the Metascape online platform. The top six GO-BP terms include carboxylic acid metabolic process, circulatory system process, and response to exogenous stimuli; GO-CC terms involve the cell body, mitochondrial matrix, and collagen-containing extracellular matrix; GO-MF terms are associated with oxidoreductase activity, protein homodimerization activity, and cell adhesion molecule binding (Figure 4A,B).

**Figure 5:**
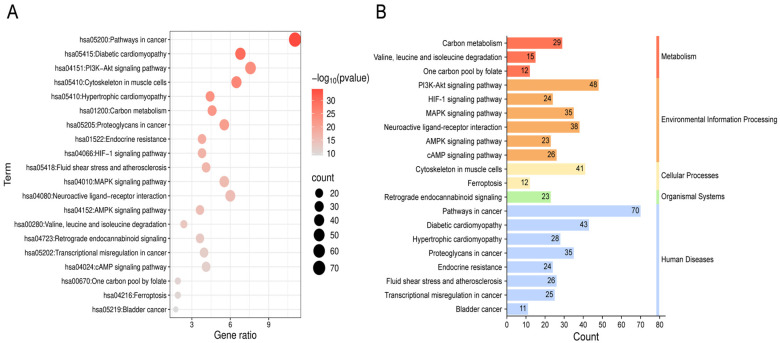
Enrichment analysis of KEGG for 633 shared genes. (**A**) The bubble chart depicted the twenty most significantly enriched KEGG signaling pathways, ranked by ascending FDR values. “hsaXXXXX” is the KEGG human pathway identifier. Gene ratio: proportion of input genes that map to the pathway. (**B**) The count denotes the number of enriched KEGG pathways in each major functional category.

A total of 180 pathways were identified through KEGG pathway enrichment analysis (adjusted *p* < 0.05). The top 20 pathways with the lowest FDR values were selected for visualization and further analysis. These pathways were depicted using bar plots and bubble charts (Figure 5A,B). The analysis primarily involved metabolic pathways such as carbon metabolism; signal transduction pathways including PI3K-Akt and MAPK pathways; disease-related pathways such as oncogenic signaling and diabetic cardiomyopathy; as well as cellular physiological pathways like cytoskeletal organization in muscle cells.

### 2.5. Molecular Docking Analysis of Core Targets of Triclosan and Neurodevelopmental Disorders

**Figure 6:**
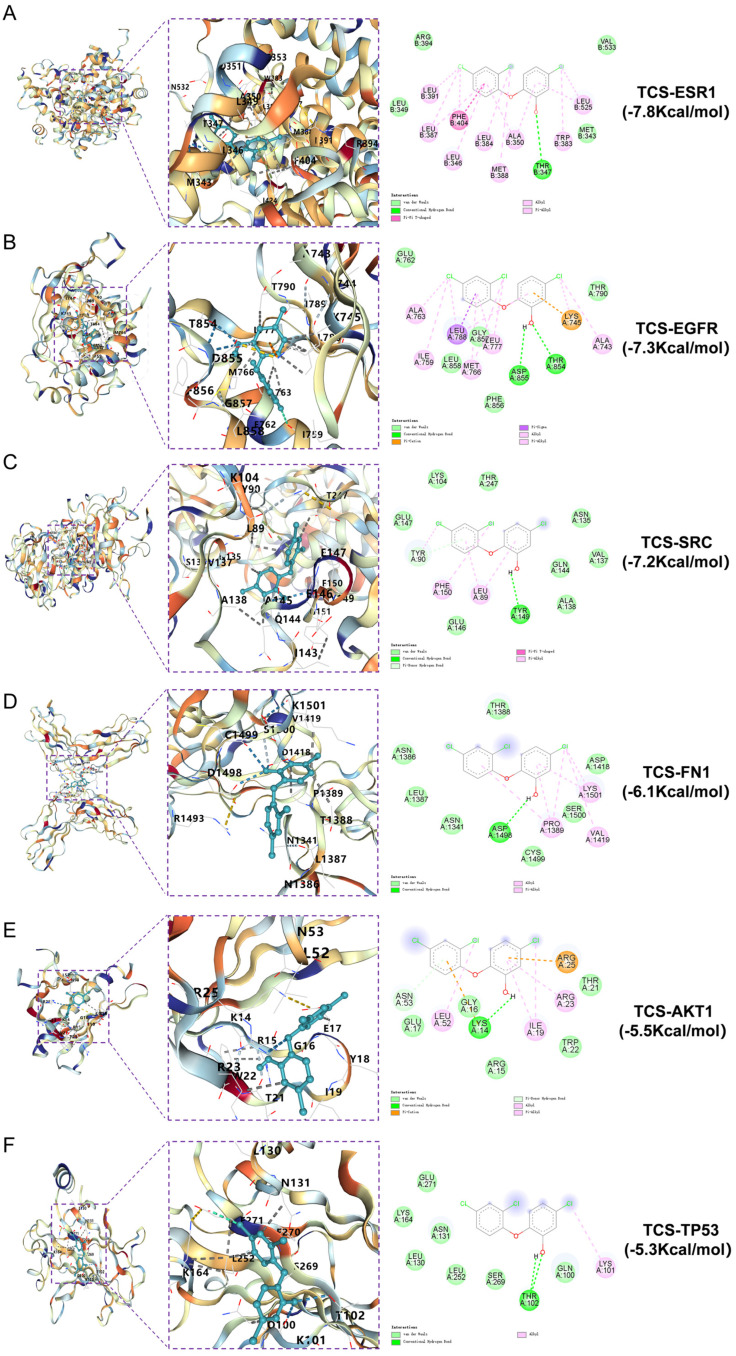
Molecular docking of triclosan with ESR1, EGFR, SRC, FN1, AKT1 and TP53, showing the lowest binding energies in both 3D and 2D formats. (**A**) TCS and ESR1; (**B**) TCS and EGFR; (**C**) TCS and SRC; (**D**) TCS and FN1; (**E**) TCS and AKT1; (**F**) TCS and TP53.

Molecular docking was performed on ESR1 (PDBID: 6SBO), EGFR (PDBID: 8A27), SRC (PDBID: 1FMK),FN1 (PDBID: 4LXO), AKT1 (PDBID: 7MYX) and TP53 (PDBID: 3D06). The CB-Dock2 results indicated binding energies of −7.8, −7.3, −7.2, −6.1, −5.5 and −5.3 kcal/mol, respectively. Binding energies below zero suggest active binding, with values less than −5.0 kcal/mol indicating strong affinity; lower values correspond to higher binding strength. The findings confirm that triclosan exhibits high affinity for these six core targets, implying a pivotal role in the molecular mechanism of triclosan-induced neurodegenerative disorders. Binding modes were visualized through 3D and 2D interaction diagrams (Figure 6).

### 2.6. Molecular Dynamics Simulation of Core Target Proteins

To further validate the binding affinity of TCS to six core targets, molecular dynamics simulations involving TCS and these targets were conducted in order of decreasing binding free energy. RMSD effectively measures the conformational stability of proteins and ligands, indicating the extent of atomic positional deviation from their initial configurations. Lower RMSD values suggest greater conformational stability [23].

**Figure 7:**
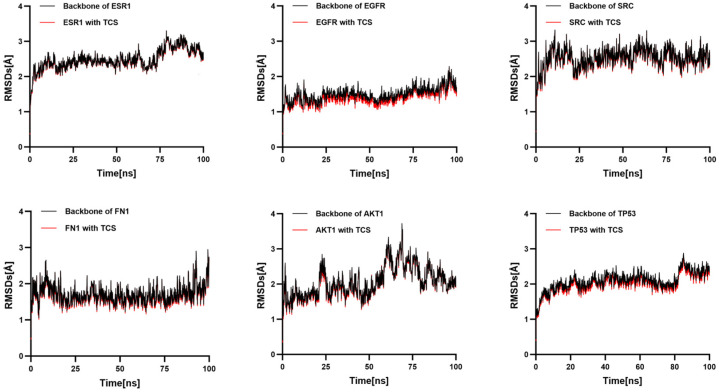
Temporal variation in RMSD values for protein–ligand complexes.

RMSD was employed to assess the stability of the simulation systems. Each RMSD plot comprises two traces: the black trace represents the fluctuation of the receptor protein’s backbone, while the red trace denotes the fluctuation of the receptor–triclosan complex’s overall backbone. The near-superimposition of these traces suggests that ligand binding does not induce significant additional conformational perturbation. The stability of the complex is primarily dictated by the protein backbone, with triclosan maintaining a relatively fixed position within the binding site. Notably, the TCS-EGFR complex exhibits relatively low RMSD fluctuations, indicating a particularly stable interaction structure, which is consistent with the molecular docking binding energy results (Figure 7).

**Figure 8:**
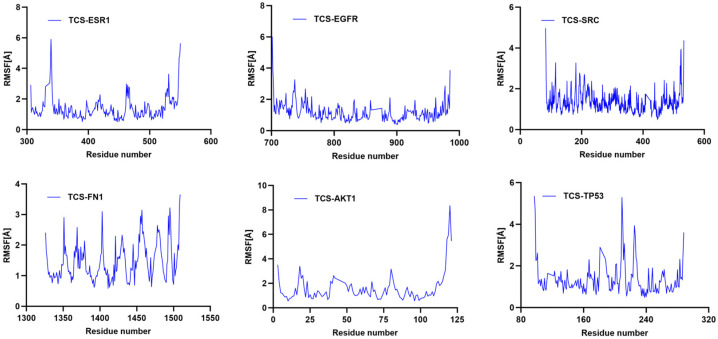
Temporal variation in RMSF values for protein–ligand complexes.

**Figure 9:**
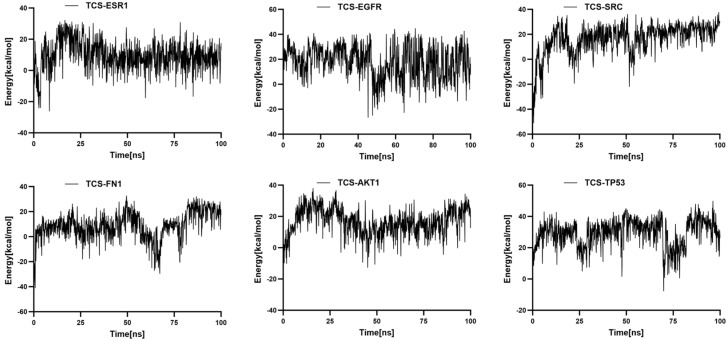
Temporal variation in binding free energy in protein–ligand complexes.

RMSF indicates the flexibility of amino acid residues within the protein [24]. Across all complexes, RMSF values for amino acid residues were relatively low (primarily between 1 and 6 Å), except at the C- and N-termini. Additionally, the energy fluctuations of the complexes formed between TCS and the six target proteins remained relatively stable (Figure 8). For example, the TCS-TP53 complex exhibited some oscillations in energy during the simulation but maintained within a specific range, indicating overall energetic stability post-binding. Similarly, the energy curves for TCS-EGFR and TCS-ESR1 complexes showed dynamic equilibrium trends with lower average energies, reflecting particularly stable binding energies with EGFR and ESR1 (Figure 9). These thermodynamic energy fluctuation data support the stability of TCS-target protein interactions, corroborating the conformational stability indicated by RMSD analysis.

In summary, aside from AKT1, five target proteins including TP53 demonstrated stable binding interactions with TCS. Notably, TCS exhibited the lowest RMSD value with EGFR, and the system remained stable and compact, suggesting that EGFR may play a key role in TCS-induced NDDs.

## 3. Discussion

This study employed network toxicology approaches to identify six core genes—*EGFR*, *TP53*, *AKT1*, *SRC*, *FN1*, and *ESR1*—highlighting their potential molecular associations with triclosan and NDDs. Notably, EGFR, a transmembrane receptor tyrosine kinase, exhibits aberrant activation that modulates neuronal survival and apoptosis via the PI3K-Akt and MAPK signaling pathways, which are closely linked to amyloid-beta deposition and tau protein phosphorylation in Alzheimer’s disease [25,26]. In this investigation, the GSE230714 dataset revealed a significant downregulation of EGFR expression. Molecular docking studies indicated a binding energy of −7.3 kcal/mol with TCS, suggesting that TCS may induce neuronal apoptosis by directly inhibiting EGFR activity and interfering with downstream neuroprotective signaling pathways. As a tumor suppressor gene, TP53 dysfunction may exacerbate neuronal DNA repair deficiencies and induce apoptosis under oxidative stress conditions, intersecting mechanistically with the dopaminergic neurodegeneration observed in Parkinson’s disease [27]; AKT1, as a central component of the PI3K-Akt signaling pathway [28], whose dysregulation can disrupt neuronal energy homeostasis, particularly in glucose uptake and mitochondrial function regulation, is critically involved [29]. KEGG pathway enrichment analysis indicating abnormalities in carbon metabolism further corroborates the disruption of this signaling cascade. These findings align with the observations of Wang et al. [17], who demonstrated that TCS induces neuronal oxidative damage by inhibiting AKT phosphorylation and attenuating the Nrf2/HO-1 antioxidant pathway.

From a pathway-level perspective, the enrichment of GO terms related to carboxylic acid metabolic processes and responses to exogenous stimuli suggests that triclosan may induce chronic neurotoxicity by disrupting neuronal metabolic homeostasis and stress response mechanisms. The association between GO-CC and the FN1 gene warrants further investigation; as a cell adhesion molecule, aberrant FN1 expression could compromise blood–brain barrier integrity or disrupt neuron-glia interaction networks [30], potentially contributing to the neuroinflammatory microenvironment observed in neurodevelopmental disorders. The KEGG-identified PI3K-Akt and MAPK signaling pathways serve not only as central convergence points for core oncogenic genes but also directly regulate critical neurobiological processes such as synaptic plasticity and cytokine secretion [31,32,33]. The enrichment of cancer-related pathways may reflect the synergistic interaction between triclosan-induced aberrant proliferative signaling and neurodegenerative mechanisms. It is noteworthy that the methodology employed in this study aligns with the work of Zhang et al. [34] concerning phthalate-induced depression. Both studies identify the PI3K-Akt pathway and endocrine resistance as central hubs in the pathogenic mechanisms of endocrine-disrupting chemicals, suggesting that various environmental pollutants may interfere with physiological functions via conserved signaling axes.

The molecular docking results further indicate that triclosan exhibits high binding affinity to six core genes, suggesting its potential to interfere with multiple biological pathways through direct targeting of these proteins. For instance, as an estrogen receptor, ESR1’s interaction with triclosan may mediate endocrine disruption via its estrogenic activity, potentially contributing to NDDs [35]. Research indicates that prenatal exposure to triclocarban disrupts ESR1-mediated signaling pathways, induces sex-specific epigenetic modifications, and perturbs the expression of neurogenesis and neurotransmitter-related genes in the postnatal murine brain [36]. Activation of SRC kinases may further facilitate neurotoxicity induced by β-amyloid through modulation of microglial activation states [37]. These mechanisms collectively delineate a complex network whereby triclosan contributes to neurodevelopmental disorder pathogenesis via a multidimensional cascade involving metabolic dysregulation, signal transduction impairment, and cellular homeostasis imbalance.

Molecular dynamics simulations confirm that EGFR is the core node with the highest binding stability among NDD-related target proteins interacting with TCS. The conformational complex formed between EGFR and TCS can perpetually activate the PI3K-Akt signaling pathway, thereby driving neuronal metabolic dysregulation and inflammatory cascades. Integrating prior network toxicology data and molecular docking results, the characteristic of EGFR’s conformational stability and sustained signaling positions it as a pivotal molecular hub in TCS-induced neurotoxicity. Future investigations should focus on functional validation of the EGFR-TCS complex to elucidate its specific impacts on synaptic plasticity and blood–brain barrier integrity, providing a theoretical basis for targeted prevention strategies against NDDs. Additionally, the observed transient binding of AKT1 with TCS suggests the necessity for further exploration of upstream regulatory modifications, aiming to refine the understanding of the multi-target neurotoxic mechanisms associated with TCS.

It is noteworthy that this study has inherent limitations. Although a potential mechanistic framework was constructed through bioinformatics analysis, there is a lack of in vivo and in vitro experimental validation of the core gene-pathway axis, particularly regarding the differential specificity of triclosan across various NDDs subtypes. Additionally, in human exposure scenarios, triclosan often co-occurs with other environmental pollutants, and the combined toxicological effects and interactions with genetic backgrounds remain to be elucidated. Future research should integrate advanced techniques such as single-cell sequencing and organoid models to further delineate the neurotoxic targets of triclosan within neuron-glia interaction networks, thereby providing more precise scientific evidence for environmental risk assessment of NDDs.

## 4. Materials and Methods

### 4.1. Triclosan Target Identification and Data Collection

The chemical structure and SMILES notation of triclosan were retrieved from PubChem (CID: 5460576). Triclosan and its structural formulas are shown in Table 1. Potential triclosan targets were identified through database searches in TargetNet, SEA, SwissTargetPrediction, and the CTD, with the species restricted to *Homo sapiens*. Target names were standardized using STRING and UniProt databases. The consolidated and deduplicated target list was used to construct the triclosan target repository.

### 4.2. Neurodevelopmental Disorder Target Identification

Utilize “Neurodevelopmental Disorders” as the search term to retrieve associated targets from GeneCards, OMIM, and DisGeNET databases. Subsequently, integrate these targets into a consolidated NDDs target dataset. Inclusion criteria encompass a relevance score of ≥10 in GeneCards and a GDA score of ≥0.4 in DisGeNET.

### 4.3. Shared Genetic PPI Network and Core Target Identification

Construct a Venn diagram using the online platform for data analysis and visualization https://www.bioinformatics.com.cn/static/others/jvenn/example.html (accessed on 7 September 2025) to visualize the intersection between triclosan targets and NDDs-associated genes, thereby identifying overlapping genes.

### 4.4. Protein–Protein Interaction (PPI) Network Construction

Import the shared gene set into the STRING database, setting parameters: species as *Homo sapiens*, interaction confidence score > 0.9 (ultra-high confidence), to generate the PPI network. Import the TSV file generated from STRING into Cytoscape version 3.10.0 (Cytoscape Consortium, La Jolla, CA, USA). Employ the CytoNCA plugin to perform network topology analysis, identify candidate genes, and compute six key parameters: Betweenness, Closeness, Degree, Eigenvector, LAC, and Network. Subsequently, identify core genes as those exhibiting all six parameter values exceeding the median.

### 4.5. Validation of Gene Expression in Randomized Samplesn

Retrieve gene expression matrices from the GEO database using the keywords “Neurodevelopmental Disorders” and “*Homo sapiens*.” Perform grouping and differential expression analysis of key target genes between normal and diseased samples utilizing the GEO2R online tool, with results visualized through volcano plots https://www.bioinformatics.com.cn/plot_enhanced_volcano_plot_138 (accessed on 7 September 2025).

### 4.6. GO and KEGG Enrichment Analysis

Utilized the Metascape database for GO functional enrichment and KEGG pathway analysis. Converted intersecting gene symbols to Entrez Gene IDs, selected *Homo sapiens* as the species, and performed enrichment analysis across GO categories (biological process, cellular component, molecular function) and KEGG pathways. Significance threshold was set at *p* < 0.05 with Benjamini–Hochberg correction. The top six GO terms and top twenty KEGG pathways were selected based on *q*-values, and the results were exported as CSV files for visualization through bioinformatics tools, generating bubble plots and bar charts https://www.bioinformatics.com.cn/?keywords=pathway (accessed on 7 September 2025).

### 4.7. Molecular Docking of Triclosan with Core Target Proteins

Molecular docking validation was performed on the CB-Dock2 online platform using triclosan as the ligand and the top six core targets based on degree values from the PPI network as receptors https://cadd.labshare.cn/cb-dock2/ (accessed on 7 September 2025) [38]. Initially, triclosan isomer SMILES were obtained from PubChem. Crystal structures of *Homo sapiens* with a resolution < 2.5 Å were downloaded from RCSB PDB based on UniProt ID. If the structure contained a co-crystal ligand, the original ligand was first removed within the platform, and redocking was performed to validate the system’s reliability with RMSD ≤ 2 Å. Subsequently, the platform automatically preprocessed the protein (hydrogenation, side-chain completion, removal of water and heteroatoms) and ligand (charge addition, 3D conformation generation). A blind docking mode was employed, and potential binding sites were identified using a curvature cavity algorithm. The binding mode with the lowest AutoDock Vina version 1.1.2 (The Scripps Research Institute, La Jolla, CA, USA) score was selected as the optimal conformation. Binding affinity was directly characterized using the binding energy (kcal/mol) output by the platform, and the interaction mode was visualized through the CB-Dock2 built-in 3D view and Discovery Studio 2019 (Dassault Systèmes BIOVIA, Vélizy-Villacoublay, France) 2D diagrams.

### 4.8. Molecular Dynamics Simulation

The receptor and ligand structures were preprocessed using YASARA version 10.3.16 (Bio-Prodict BV, Nijmegen, The Netherlands),which included the removal of irrelevant atoms, the addition of hydrogen atoms, and the assignment of protonation states (pH = 7.4) to optimize the structure. Given that the PDBQT atom types and partial charges generated by CB-Dock2 could not be recognized by YASARA’s AMBER force field, we re-executed molecular docking within YASARA, while maintaining the search grid and exhaustiveness, to screen for the complex with the best binding energy and reasonable conformation as the target system. Subsequently, based on the AMBER force field, the complex was placed in an explicit solvent environment, and a cubic simulation box was established using periodic boundary conditions (“around-all-atoms” mode + 5 Å buffer, α = β = γ = 90°); water and ions were added according to the software’s default settings. After the system was subjected to energy minimization and equilibration, a 100 ns (100,000 ps) molecular dynamics simulation was performed, with real-time recording of parameters such as RMSD, energy, and RMSF. Post-simulation, the resulting data were visualized using GraphPad Prism version 9.0 (GraphPad Software, San Diego, CA, USA), with line graphs generated to facilitate intuitive interpretation of the analysis outcomes.

### 4.9. Data Sources

All databases were accessed between April and September 2025. The specific versions of these databases are shown in Table 2.

## 5. Conclusions

This study employed network toxicology and molecular docking analyses to comprehensively evaluate the neurodevelopmental hazard potential of triclosan, identifying 633 intersecting targets associated with triclosan-induced NDDs. The mechanistic pathways involve endocrine disruption (including estrogenic activity), enhancement of glycolytic flux via PI3K-Akt pathway interference affecting neuronal energy metabolism, and regulation of synaptic plasticity and cytokine release. Molecular docking results of the top six core targets—EGFR, TP53, AKT1, SRC, FN1, and ESR1—selected through median-based algorithms, indicate their pivotal roles in mediating triclosan-induced NDDs. Molecular dynamics simulations confirm that EGFR exhibits the highest binding stability among NDDs-related targets, with the triclosan–EGFR complex conformation capable of sustained activation of the PI3K-Akt pathway, thereby driving neuronal energy dysregulation and inflammatory cascades. While regulatory assessments of environmental endocrine disruptors like triclosan predominantly focus on acute toxicity and carcinogenicity, this research uniquely applies a multidimensional systems biology approach to elucidate the molecular mechanisms underlying triclosan-induced NDDs, providing a methodological framework for the risk assessment of emerging environmental contaminants on neurodevelopment.

## Figures and Tables

**Table 1:** The chemical formula and structural formula of triclosan.

Smile	Structure
C1=CC(=C(C=C1Cl)O)OC2=C(C=C(C=C2)Cl)Cl	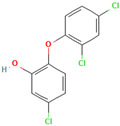

**Table 2:** Data source and access link.

Database Name	Access Address
PubChem	https://pubchem.ncbi.nlm.nih.gov/
TargetNet	http://targetnet.scbdd.com/calcnet/index/
SwissTargetPrediction	http://www.swisstargetprediction.ch/
CTD	http://ctdbase.org/
GeneCards	https://www.genecards.org/
OMIM	https://omim.org/
DisGeNET	https://www.disgenet.org/
UniProt	https://www.uniprot.org/
STRING	https://string-db.org/
GEO	https://www.ncbi.nlm.nih.gov/geo/
Metascape	https://metascape.org/
RCSB PDB	https://www.rcsb.org/

## Data Availability

The original contributions presented in this study are included in the article. Further inquiries can be directed to the corresponding author.

## Supplementary Materials

The following supporting information can be downloaded at: https://www.mdpi.com/article/10.3390/ijms26199458/s1.
